# Exploring the prioritisation of sleep, diet, and physical activity as pillars of health: correlates and associations with health behaviours in Australian adults

**DOI:** 10.1186/s44167-023-00035-3

**Published:** 2023-12-02

**Authors:** Charlotte C Gupta, Mitch J Duncan, Sally A Ferguson, Amanda Rebar, Corneel Vandelanotte, Madeline Sprajcer, Saman Khalesi, Lauren A. Booker, Caroline Rampling, Gabrielle Rigney, Grace E Vincent

**Affiliations:** 1 Appleton Institute, School of Health, Medical and Applied Sciences, Central Queensland University, Adelaide, South Australia Australia; 2 School of Medicine & Public Health, Faculty of Health and Medicine, The University of Newcastle, University Drive, Callaghan, NSW Australia; 3 Active Living Research Program, Hunter Medical Research Institute, New Lambton Heights, NSW Australia; 4 Institute for Breathing and Sleep, Austin Health, Heidelberg, VIC Australia; 5 La Trobe Rural Health School, La Trobe University, Bendigo, VIC Australia

**Keywords:** Sleep, Activity, Food, Shiftwork, Prioritise, Health

## Abstract

**Background:**

Chronic disease is the leading cause of death globally. Sleep, diet, and physical activity are modifiable health behaviours that are key for reducing the burden of chronic disease. These health behaviours are collectively termed ‘The 3 Pillars of Health’ and are critical for populations who are at risk of poor health. Shiftworkers are one such at-risk population. To target behavioural change it is critical to first understand which of these health behaviours Australians currently prioritise. Therefore, this study aimed to investigate how Australians (including shift workers) prioritise sleep, physical activity, and diet, and examine the associations with correlates of health behaviours.

**Methods:**

Two cohorts of Australian adults were sampled. A cohort of 1151 Australian adults (54% female, aged 18–65 years) including employed (in any work schedule), unemployed, studying, and retired completed a phone interview. A cohort of 533 Australian shiftwork-only adults (76% female, 18–72) completed an online survey. All participants were asked which health behaviour (sleep, physical activity, or diet) they prioritised in their own life. Behavioural correlates of sleep, diet, and physical activity (sleep duration, frequency of moderate to physical activity, healthy dietary behaviour), and years of shiftwork experience were also collected. Multinomial logistic regressions were used to investigate the association between the highest prioritised pillar of health and the behavioural correlates.

**Results:**

Diet was prioritised by the Australian adults (49%), whereas sleep was prioritised by the shiftwork-only sample (68%). Australian adults who prioritised diet were significantly more likely to report diets with less fast-food consumption (*p* < 0.002) and more fruit consumption (*p* < 0.002) compared to those that prioritised sleep. For the shiftwork-only sample, those with 16–30 years of shiftwork experience were significantly more likely to prioritise sleep compared to diet (*p* < 0.05). However, prioritising sleep was not associated with meeting the sleep duration recommendations in the shiftwork-only sample.

**Conclusions:**

Across two cohorts of Australians, prioritisation of health behaviour was only associated with actual behaviour for diet. This may reflect different motivations for prioritising different health behaviours, in addition to different capabilities to change different health behaviours. Future research should include longitudinal methodologies to understand how behaviour prioritisation changes over work- and life-span, and any associations with actual health behaviour.

**Supplementary Information:**

The online version contains supplementary material available at 10.1186/s44167-023-00035-3.

## Background

Chronic diseases, such as heart disease, diabetes, and obesity, are the leading cause of death globally [1, 2]. A strategy to reduce the burden of chronic disease is to target modifiable health behaviours such as smoking, nutrition, alcohol consumption, physical activity, sitting and sleep [3–5]. Current perspectives for health behaviours focus on ‘The Pillars of Health’ [3, 6, 7]. The behaviours included as pillars of health vary in the literature, but the most common are physical activity, diet, sleep, smoking, alcohol use, and relaxation [3, 6, 7]. These health behaviours are also promoted in public policy, for example, the American Heart Foundation’s ‘Life’s Essential 8’ [8], which champions many of the pillars including sleep, physical activity, and diet. Of the pillars of health represented in research and policy, sleep, physical activity, and diet are commonly referred to as ‘The Three Pillars of Health” as they are essential for life, impact each other, and are associated with a number of health outcomes [6, 9–13].

Inadequate sleep [14], insufficient physical activity [15], and an unhealthy diet [16] are each independently associated with an increased risk of chronic disease and therefore independently contribute to a large burden on the healthcare system. In 2016–2017 the healthcare costs associated with inadequate sleep in Australia were estimated to be almost $1.8 billion (AUD) [17], the economic impact attributable to inadequate consumption of fruit and vegetables results in total costs of over $2 billion (AUD) [18], and the annual healthcare and lost productivity costs associated with physical inactivity are in excess of $1.6 billion (AUD) [19]. Important components of adequate sleep, diet, and physical activity include obtaining 7–9 h sleep per night [20], meeting the Australian recommended serves of fruits (> 2 per day) and vegetables (> 5 per day) [21], and obtaining at least 150 min of physical activity per week at moderate-to-vigorous intensity [1].

The risk of chronic disease can also be heightened based on population-level differences, such as work schedule [22, 23]. For example the risk of chronic disease is greater in shiftworkers, that is, anyone working outside of conventional daytime hours (e.g., early morning, late evening, night-work, and on-call) [22, 24], compared to day workers. Shiftwork leads to circadian disruption, and is associated with altered sleep-wake patterns and disruptions to other health behaviours [22, 24–26], such as altered patterns of food intake [27], reduced physical activity [28, 29], and increased likelihood of smoking [30]. Consequently, shiftworkers are up to five times more likely to experience a coronary event than non-shift workers [31], as well as being at an increased risk of diabetes, high cholesterol and cardiovascular disease [32, 33]. Additionally, shiftworkers experience unique external barriers to sleep, diet, and physical activity that are important to consider. For example, external barriers such as hospital cafeterias being closed at night influences the food that shiftworkers can consume on shift [27], and a lack of a quiet sleeping environment at work can influence whether sleep is prioritised on shift [74]. As with the general population, facilitators to health behaviours can include workplace initiatives, clear focus on health benefits, and peer support [27, 64, 74].

Sleep, diet, and physical activity are independently associated with chronic disease risk, yet they are behaviours that do not occur independently and indeed engagement in one behaviour influences engagement in other behaviours [34]. For example, there is a bidirectional relationship between physical activity and sleep, such that increased physical activity is associated with better sleep and, conversely, poor sleep is associated with reduced physical activity [35–39]. Further, increasing physical activity is more effective for improving health outcomes when an individual also obtains adequate sleep [40]. Sleep quality and quantity can also be influenced by dietary behaviours [41, 42], and dietary behaviours can be influenced by sleep [43]. There is also evidence that engaging in all three behaviours is beneficial to health [44, 45]. For example, individuals who eat a healthy diet tend to engage in physical activity and get adequate sleep, and have lower risk of chronic disease [46]. Interventions that consider the influence of multiple behaviours (sleep, diet, and physical activity) may be important for reducing the burden of chronic disease risk.

In general, effective behaviour change at the individual level requires motivation, capability, and opportunity (as per the COM-B model of behaviour) [47]. Some evidence suggests that targeting multiple behavioural factors together is beneficial for the success of an intervention [48]. For example, it may be that if a person has sufficient motivation for changing one behaviour, this motivation can generalise to a motivation for becoming healthier overall. In this case, the regulation skills utilised to change one behaviour, such as planning and problem solving, could be applied to other behaviours [4]. However, while the skills needed to change health behaviours may be transferable, behaviour change, be it for one or multiple behaviours, is challenging especially as motivation wanes and priorities shift [49]. A lack of time and competing priorities (e.g. work-life conflict) are the most common reasons given for not prioritising health behaviours [50]. Therefore, if people are not engaging in any health behaviours, a change to three behaviours at once may not be feasible [46]. As a first step, consideration of how people prioritise behaviours should be explored. This is necessary for capturing a snapshot of current behaviours and attitudes to gauge the level of motivations and helps act as a starting point for interventions to change subsequent behaviours.

It is currently unknown how Australian adults prioritise the pillars of health (sleep, diet, and physical activity) and if prioritisation is related to how they engage in actual health behaviours. Further, we do not know if cohorts with different levels of chronic disease risk and work demands to the general public, e.g., shiftworkers, prioritise the pillars differently. Therefore, the current study investigated in a general population cohort, and shiftworking cohort: (a) what pillars of health Australians prioritise, and (b) what socio-demographic and health behaviour correlates are associated with this health behaviour prioritisation.

## Method

### Design

This paper presents data from two cross-sectional studies: Study 1 was a cohort of Australian adults, including employed (in any work schedule), unemployed, studying, and retired, and Study 2 was a cohort of current Australian shiftworkers. A STROBE (cross-sectional) checklist guides the reporting of both studies.

Study 1 uses data from the National Social Survey, a descriptive cross-sectional survey conducted by the Population Research Laboratory at CQUniversity. This survey comprised of core information assessing demographics, health behaviours, chronic diseases status, and quality of life. The current study analyses data from a subset of the questions from the 2017 (July-August) National Social Survey. Data from the questions referring to sleep duration have been previously published [51, 52]. All participants provided verbal informed consent prior to the start of the survey. Ethics was obtained from the CQUniversity Human Research Ethics Committee (H14/09-203).

Study 2 uses from a cross-sectional survey on the sleep hygiene knowledge of Australia shiftworkers conducted in June-August 2021. This survey comprised of questions on core demographics, sleep hygiene knowledge, sleep hygiene practices, sleep quantity, and sleep quality. Data from the questions referring to sleep hygiene have been published elsewhere [26]. The current study includes data from questions on the prioritisation of health behaviours, sleep quality, and sleep quantity. All participants provided informed consent and the study was granted ethics approval by the CQUniversity Human Research Ethics Committee (2021-03).

## Participants

### Study 1

All states and territories in Australia were sampled using randomly selected landlines from an Australian database and dual frame random digit dialling of phones to conduct computer-assisted-telephone-interviewing. For mobile telephone numbers, the respondent was the person who received the phone call and for landline telephone numbers, a respondent within each household was selected using a process to ensure an equal yet random selection of female and male participants. All participants were verbally told the purpose of the study and asked if they were aged 18 years or older and resided in Australia to ensure eligibility.

### Study 2

Australian shiftworkers from all states and territories were eligible to participate in the online survey from June 2021 to August 2021. Online advertisements were posted on social media (Facebook and Twitter) and through convenience sampling, flyers were sent to companies that employed shiftworkers. Additional recruitment was conducted using convenience sampling and snowballing methods. Inclusion criteria included: self-identified as a current shiftworker, an Australian resident, and 18 years or older.

## Procedure

### Study 1

The research team at the Population Research Laboratory at CQUniversity conducted the interviews, after training in the computer-assisted telephone interviewing system. Interviews lasted approximately 40 min and were conducted at various times throughout the day and evening, seven days per week. Upon making contact, interviewers identified themselves and then asked screening questions to select the respondent. If the interviewers did not establish contact on their first call, a maximum of five call back attempts were made. No renumeration was provided.

### Study 2

Participants were provided a link to the online survey, hosted on Qualtrics [53]. The survey took approximately 20 min to complete. Participants were informed that the survey was anonymous and voluntary, and no remuneration was provided. At the conclusion of the survey participants were provided with a link to a separate survey, also hosted on Qualtrics, where they had the option of providing their email address to receive a plain English summary of their results.

## Measures

### Both studies

#### Prioritising health behaviours

In Study 1 and 2, participants were asked to prioritise the three health behaviours of interest (‘The Pillars of Health’; sleep, diet, and physical activity). Participants were asked, ‘Which of the following most accurately reflects the order of importance for optimal health and wellbeing in your own life?’ with 6 options ordered from most to least important (1. sleep, diet, physical activity; 2. sleep, physical activity, diet; 3. diet, sleep, physical activity; 4. diet, physical activity, sleep; 5. physical activity, sleep, diet; 6. physical activity, diet, sleep).

#### Sleep

Sleep duration was assessed by asking “Thinking about a usual weekday, how much sleep do you regularly get?”. Response options were < 1 h, 1–2 h, 2–3 h, 3–4 h, 5–6 h, 6–7 h, 7–8 h, 8–9 h, 9–10 h, 10–11 h, 11–12 h, or > 12 h. Participants were categorised based on the National Sleep Foundation sleep recommendations for adults [54], which is 7–9 h of sleep per night. Therefore, sleep duration per night was coded into a categorical variable with two categories: meet recommendation (≥ 7 h - ≤9 h sleep) or does not meet the sleep recommendations (< 7 h - >9 h sleep). Participants were also asked about sleep disorders with the question ‘Do you currently suffer from any diagnosed, chronic (> 6 months) sleep-related disorder?’ with response options: sleep apnoea, snoring, insomnia, restless legs, another sleep-related disorder, a health condition that impacts sleep, or none. This was coded into a categorical variable: sleep disorder or no sleep disorder.

In Study 2, sleep duration was assessed by asking ‘how many hours of actual sleep do you get at bedtime?’. Participants were categorised by the National Sleep Foundation sleep recommendations for adults [54], which is 7–9 h of sleep per night. Therefore, sleep duration per night was coded as a categorical variable with two categories: meet recommendation (≥ 7 h - ≤9 h sleep) or does not meet the sleep recommendations (< 7 h or > 9 h sleep). Participants in Study 2 were also asked about sleep disorders, with the question, ‘Do you currently suffer from any diagnosed sleep-related disorders?’ with response options: sleep apnoea, snoring, insomnia, restless legs, another sleep-related disorder, a health condition that impacts sleep, or none. This question was also coded into a categorical variable: sleep disorder or no sleep disorder.

### Study 1

#### Sociodemographic questions

Sociodemographic characteristics included gender (male, female, non-binary/prefer not to say), age (a continuous variable which was coded to a categorical variable with 4 levels: 18–34 years, 35–44 years, 45–54 years, > 55 years), and current work status (employed or not employed). Participants also self-reported height and weight, which were used to determine Body Mass Index (BMI; kg/m^2^). BMI categories were coded as underweight-normal (≤ 24.9 kg/m^2^), and overweight-obese (> 25.0 kg/m^2^). Detail on other sociodemographic characteristics measured in the National Social Survey but not included in the current analyses are published elsewhere [52, 55, 56].

#### Diet

To assess dietary behaviours, three aspects of a healthy diet were asked about: fruit, vegetable, and fast-food consumption. To assess fruit consumption, participants were asked “How many serves of fruit do you eat on a usual day? One serve of fruit is equivalent to one medium piece or two small pieces of fruit.” This was a continuous variable coded into a categorical variable: meeting the daily recommendations for fruit intake [21] (≥ 2 serves per day) or not meeting the daily recommendations for daily fruit intake (< 2 serves per day). To assess vegetable consumption, participants were asked ‘How many serves of vegetables do you eat on a usual day? One serve of vegetables is equivalent to half a cup of cooked vegetables or one cup of salad vegetables.’ This was a continuous variable, coded into a categorical variable: meeting the daily recommendations for vegetable intake [21] (≥ 5 serves per day) or not meeting the daily recommendations for daily fruit intake (< 5 serves per day). Fast-food consumption was assessed by asking ‘In the last week (the last 7 days), how many times did you eat something from a fast-food restaurant like McDonald’s, Hungry Jacks, KFC, etc? This also includes other fast-food and takeaway such as fish and chips, Chinese food, and pizza for example.’ Response options included: never, once, 2–3 times, 4–5 times, 6–7 times, more than 7 times. This was coded into a categorical variable: ≤ once a week or ≥ twice a week (based on previous research indicating that fast food consumption twice a week or more is associated with poorer health outcomes than once a week or less [57, 58]).

#### Physical activity

Physical activity was assessed via The Active Australia Survey which comprises eight items to assess the frequency and duration of walking, moderate and vigorous leisure physical activity, and vigorous gardening in the past 7 days. A combined total of moderate and vigorous physical activity (MVPA) was calculated as per previous research [59, 60]. Time spent doing vigorous gardening was not included in the calculations of vigorous physical activity, consistent with Active Australia Survey recommendations [61]. As the minimum recommendation for physical activity is 150 min per week [1], the MVPA variable was dichotomised into those that met this recommendation and those that did not. This aligns with Active Australia Survey data treatment recommendations [61].

### Study 2

#### Sociodemographic questions

Demographic questions included gender (male, female, non-binary/prefer not to say); age (a continuous variable which was coded to a categorical variable with 4 levels: 18–34 years, 35–44 years, 45–54 years, > 55 years); and length of employment in shift work (a continuous variable that was coded into a categorical variable with 4 levels: 1–5 years, 6–15 years, 16–30 years, 30 + years). Participants were also asked what industry they were employed in, with response categories based on the Australian and New Zealand Standard Industrial Classification (ANZSIC; [62]) categories (mining, manufacturing, retail trade, accommodation and food services, transport, postal and warehousing, information media and telecommunications, public administration and safety, healthcare and social assistance, arts and recreation services, and government or defence). Questions on diet and physical activity were not included in study 2 as they were not part of the primary aims of the larger study which focussed on knowledge of sleep hygiene in shiftworkers [26].

### Statistical analyses

Descriptive frequency analysis was used to report sociodemographic and health characteristics, and proportion of participants that prioritised each of the health behaviours first. All analyses were conducted using Jamovi 1.2.2 (The Jamovi Project, 2021).

For Study 1, multiple logistic regression models were used to examine the associations between prioritising each health behaviour first (i.e., sleep, diet, or physical activity), with correlates of sleep, diet, and physical activity (fruit consumption, vegetable consumption, fast food consumption, sleep duration, sleep disorders, and MVPA). The reference group was determined by which health behaviour was prioritised first by a majority of participants. For Study 1, this was diet. Due to the large number of comparisons, Bonferroni correction was applied where the critical *p* value was divided by the number of comparisons (0.05/21) to result in a new critical *p* value of *p* < 0.002. Significant differences (*p* < 0.05) as a function of demographic factors (age, gender) were tested for each of the outcome variables (fruit consumption, vegetable consumption, fast food consumption, sleep duration, sleep disorders, vigorous activity, and moderate activity) using Chi square analyses. Any demographic factors that were found to be associated with an outcome variable through the Chi square analyses were entered into the adjusted multinomial logistic regressions as a covariate. Age was significantly associated with fruit consumption, fast food consumption, sleep duration, and sleep disorders, and was included as a covariate for those analyses. Gender significantly associated with fruit consumption, vegetable consumption, fast food consumption, sleep disorders, and MVPA and was included as a covariate for those analyses.

For Study 2, multiple logistic regression models were used to examine the associations between prioritising each health behaviour first (i.e., sleep, diet, or physical activity), and sleep duration, shiftwork duration, and sleep disorders. The reference group was prioritising sleep first as this behaviour was prioritised first by a majority of the sample. The critical *p* value was kept at *p* < 0.05. Significant differences (*p* < 0.05) as a function of demographic factors (age, gender) were tested for each of the outcome variables (sleep duration, shiftwork duration, and sleep disorders) using Chi square analyses. Age and gender were significantly associated with sleep duration, shiftwork duration, and sleep disorders, and as such, were included as covariates for each multinomial regression.

Findings from Study 1 and Study 2 are presented separately below.

## Results

### Participants

A total of 1265 participants completed the 2017 National Social Survey and were included in Study 1. This was a response rate of 24%, which is comparable to previous National Social Surveys in 2015 and 2016 [55]. Of these 1265 participants, 114 either did not answer or responded “I don’t know” to the prioritisation question. Therefore, the final sample included in analyses was 1151 participants. As can be seen in Table 1, a majority of the sample in Study 1 was female (52.7%), over 55 years (54.8%), had a BMI > 25kgm^2^ indicating overweight-obese classification (62.3%), no diagnosed sleep disorder (69.8%), and employed (54.5%).

A total of 748 participants completed the online survey in Study 2. Of these 748 participants, 159 only answered the demographic questions, 55 did not complete the behaviour prioritisation question, and 1 participant was from a location outside of Australia and so was excluded from the sample. The final sample consisted of a total of 533 participants, with a majority female (76.2%), aged 18–34 years (52.5%), no diagnosed sleep disorder (52.5%), 6–15 years of shiftworking experience (42.6%), and a majority (64.4%) employed in the health care and social assistance industry.

**Table 1:** Demographic characteristics of participants in Study 1 and Study 2

	Australian Adults (Study 1) (*n* = 1151)		Australian Shiftwork-only (Study 2) *(n* = 533)	
Variable	*n*	%	*n*	%
Gender				
Males	533	46.3	123	23.1
Females	618	53.7	406	76.2
Non-Binary/Prefer not to say			4	0.7
Age				
18–34	223	19.4	280	52.5
35–44	138	12.0	117	21.9
45–54	166	14.4	92	17.3
55+	624	54.2	44	8.3
BMI				
Underweight-normal	498	43.3		
Overweight-obese	653	56.7		
Diagnosed sleep disorder				
Yes	346	30.1	175	32.8
No	805	69.9	358	67.2
Employed				
Yes	639	55.5		
No	509	44.2		
No response	3	0.3		
Shiftwork duration				
1–5 years			188	35.3
6–15 years			227	42.6
16–30 years			88	16.5
30 + years			30	5.6
Shiftwork Industry				
Health care and Social assistance			343	64.4
Mining			111	20.8
Transport, postal and warehousing			29	5.4
Government or Defence			25	4.7
Manufacturing			10	1.9
Accommodation and Food services			7	1.3
Public administration and Safety			6	1.1
Information media and telecommunications			1	0.2
Retail trade			1	0.2
Behaviour Prioritised First				
Sleep	336	29.2	363	67.7
Diet	567	49.3	106	20.3
Physical activity	248	21.5	64	12.0

*Note.* For Study 1, questions on shiftwork duration and shiftwork industry were not included. For Study 2, questions on BMI and employment status were not included

### Australian adults (study 1)

**Fig. 1:**
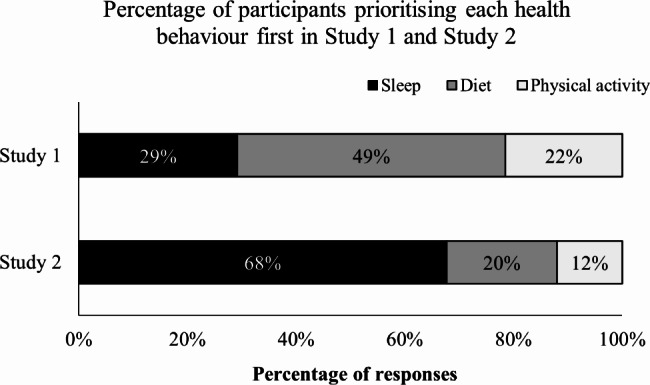
Percentage of participants in Study 1 (*n* = 1151) and Study 2 (*n* = 533) who prioritised each of the health behaviours (sleep, diet, physical activity)

In Study 1 diet was prioritised first by the majority of participants (49.3%, *n* = 567; Fig. 1), sleep was prioritised first by 29.2% of participants (*n* = 336), and physical activity was prioritised first by the least number of participants (21.5%, *n =* 248).

Diet was prioritised first by both employed (46%, *n =* 297) and unemployed (53%, *n =* 269) participants.

**Fig. 2:**
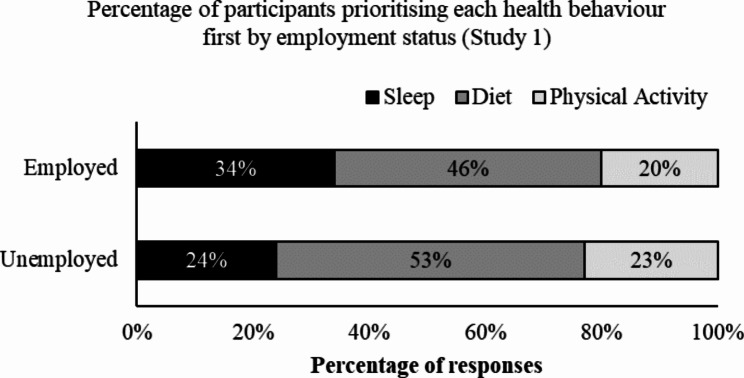
Percentage of participants in Study 1 (*n* = 1151) who prioritised each of the health behaviours (sleep, diet, physical activity) split by employment status (employed, unemployed)

**Fig. 3:**
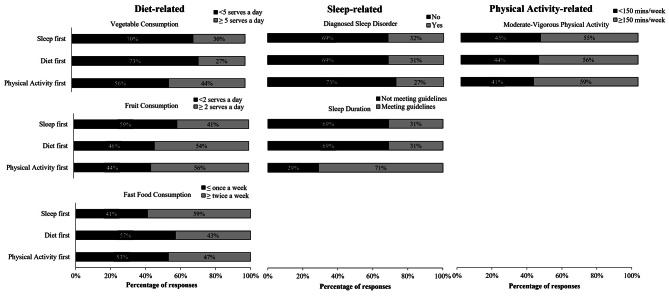
Stacked bar graphs showing the percentage of participants in Study 1 (n = 1151) who prioritised each of the health behaviours (diet, sleep, physical activity (PA)) first for each behavioural correlate (vegetable consumption, fruit consumption, fast food consumption, sleep disorder, sleep duration, moderate-vigorous physical activity)

A majority of the Australian adults in Study 1 did not have sleep disorders (69.9%), did not meet the recommended sleep guidelines (70.3%), were categorised as reporting < 5 serves of vegetables per day (86.7%), reporting ≥ 2 serves of fruit per day (50.8%), and met the recommendation for more than 150 min of moderate- to vigorous-intensity physical activity per week (56.6%). Results of the multinomial logistic regressions demonstrated that, compared to those that prioritised diet first, those that prioritised sleep first were significantly less likely to be in the category of ≥ 2 serves of fruit per day, and significantly more likely to be in the category of ≥ 2 serves of fast food per week (Table 2; Fig. 3). There were no significant associations between those that prioritised diet first and those that prioritised physical activity first.

**Table 2:** Multinomial logistic regression analysis for Australian adults (Study 1)

	All	Diet Prioritised First	Sleep Prioritised First		Physical Activity Prioritised First	
	n = 1151	n = 567	n = 336	Compared to Diet First	n = 248	Compared to Diet First
	n (%)	n (%)	n (%)	RRR (95% CI)	n (%)	RRR (95% CI)
Diagnosed sleep disorder						
Yes	346 (30.1)	178 (31.4)	106 (31.5)	1.05 (0.78–1.42)	67 (27.0)	0.86 (0.62–1.18)
No (Ref)	805 (69.9)	389 (68.6)	230 (68.5)	1	181 (73.0)	1
Sleep duration						
Meeting guidelines (7–9 h of sleep)	342(29.7)	171 (30.2)	100 (29.8)	0.98 (0.73–1.32)	71 (28.6)	1.07 (0.77–1.49)
Not meeting guidelines (< 7 or > 9 h of sleep) (Ref)	809 (70.3)	396 (68.6)	236 (68.5)	1	177 (28.6)	1
Vegetable consumption						
< 5 serves a day	991 (86.7)	485 (85.5)	294 (87.5)	0.84 (0.56–1.25)	212 (85.5)	1.06 (0.69–1.63)
≥ 5 serves a day (Ref)	160 (13.9)	82 (14.5)	42 (12.5)	1	36 (14.5)	1
Fruit consumption						
< 2 serves a day	566 (49.2)	259 (45.7)	197 (58.6)	0.62 (0.47–0.82) **	110 (44.4)	1.09 (0.81–1.48)
≥ 2 serves a day (Ref)	585 (50.8)	308 (54.3)	139 (41.4)	1	138 (55.6)	1
Fast food consumption						
≤ once a week	594 (51.6)	325 (57.3)	138 (41.1)	1.70 (1.27–2.27) **	131 (52.8)	1.14 (0.83–1.57)
≥ twice a week (Ref)	557 (48.4)	242 (42.7)	198 (58.9)	1	117 (47.2)	1
Moderate-vigorous-intensity physical activity						
< 150 min/week	500 (43.4)	248 (43.7)	151 (44.9)	0.96 (0.74–1.25)	101 (40.7)	1.10 (0.81–1.49)
≥ 150 min/week (Ref)	651 (56.6)	319 (56.3)	185 (55.1)	1	147 (59.3)	1

RRR: Relative Risk Ratio; **p < 0.002

### Australian shiftwork-only (study 2)

**Fig. 4:**
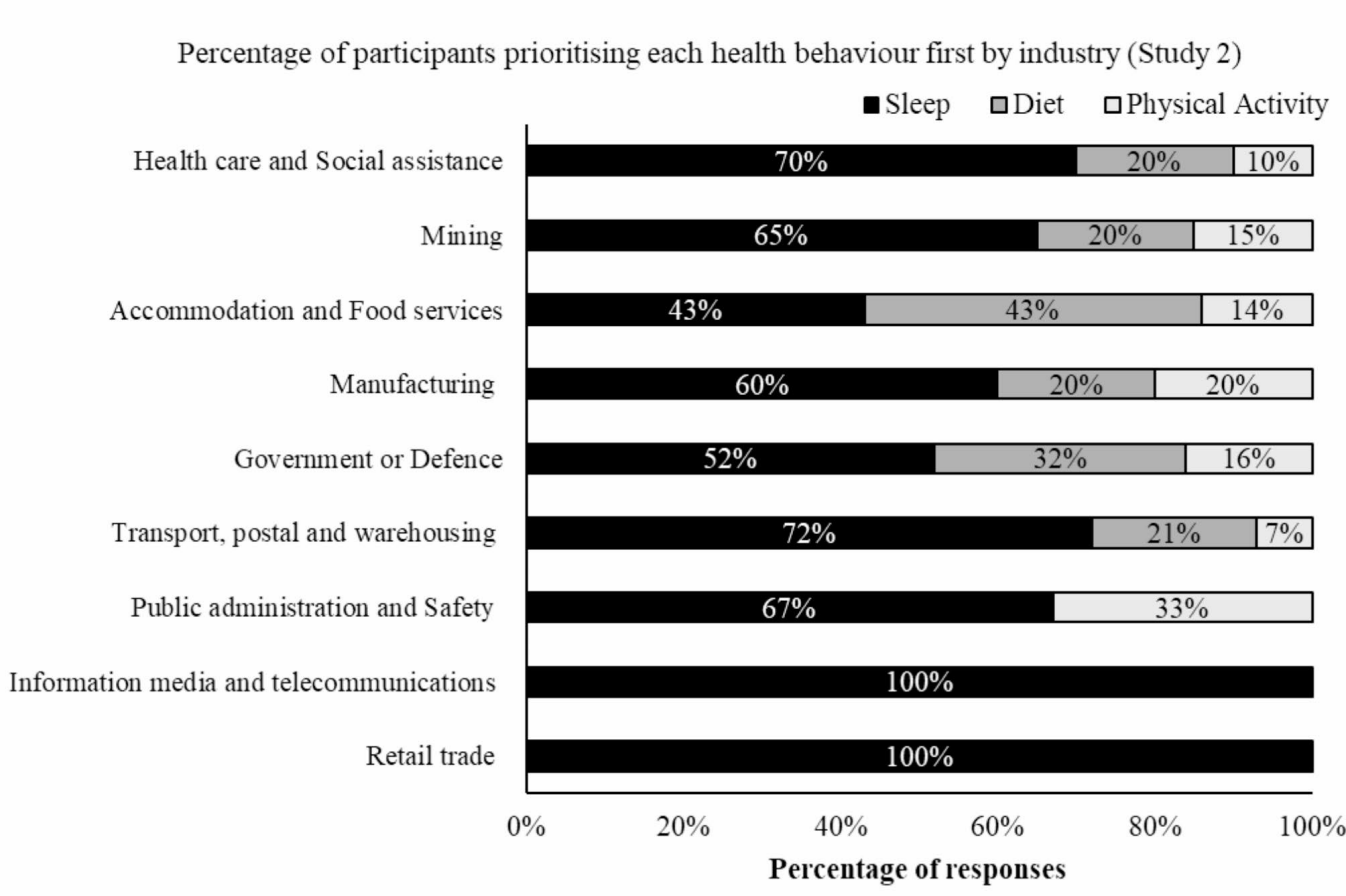
Percentage of participants in Study 2 (*n* = 533) who prioritised each of the health behaviours (sleep, diet, physical activity) in each shiftworking industry

In Study 2, sleep was prioritised first by the majority of participants (67.7%, *n* = 361; Fig. 1), diet was the second highest (20.2%, *n* = 108), and physical activity was prioritised by the least number of participants (12.0%, *n* = 64). Sleep was prioritised first by participants in each of the industry groups (Fig. 4).

**Fig. 5:**
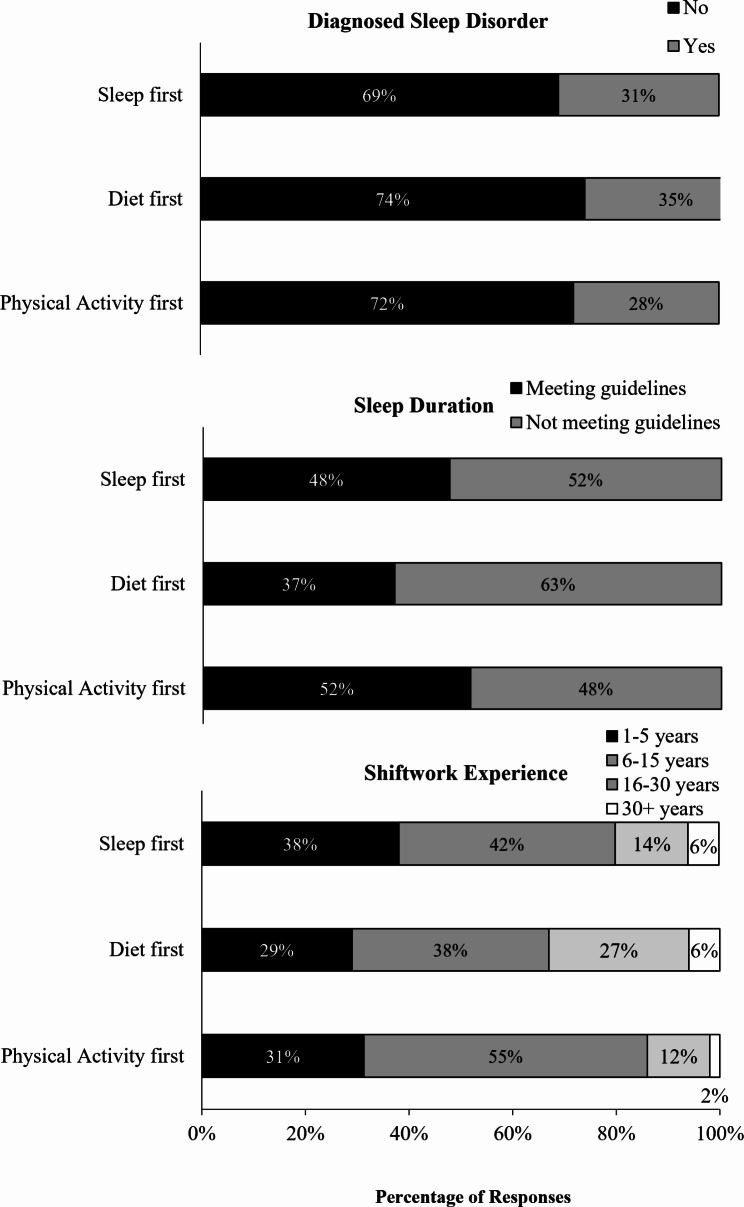
Stacked bar graphs showing the percentage of participants in Study 2 (*n* = 533) who prioritised each of the health behaviours (sleep, diet, physical activity) first for each behavioural correlate (sleep disorder, sleep duration, shiftwork experience)

A majority of the participants did not report sleep disorders (68.4%), did not meet the recommended sleep duration guidelines (54.0%), and reported 6–15 years of shiftwork experience (42.6%). Only 5.6% of participants reported more than 30 years of shiftwork experience. Results of the multinomial logistic regressions revealed that, compared to those that prioritised diet first, those who prioritised sleep first were significantly more likely to report 16–30 years of shiftwork experience (Table 3; Fig. 5). There were no other significant associations.

**Table 3:** Multinomial regression analysis for Australian shiftwork-only participants (Study 2) for associations between behavioural correlates and prioritising the health behaviours first compared to sleep

	All	Sleep Prioritised First	Diet prioritised First		Physical Activity Prioritised First	
	n = 533	n = 361	n = 108	Compared to Sleep First	n = 64	Compared to Sleep First
	n (%)	n (%)	n (%)	RRR (95% CI)	n (%)	RRR (95% CI)
Diagnosed sleep disorder						
Yes	168 (31.5)	112 (31.0)	38 (35.2)	0.89 (0.56–1.41)	18 (28.1)	0.80 (0.41–1.59)
No (Ref)	365 (68.4)	249 (69.0)	80 (74.1)	1	46 (71.9)	1
Sleep duration						
Meeting guidelines (7–9 h of sleep)	245 (46.0)	172 (47.6)	40 (37.0)	0.64 (0.41–1.01)	33 (51.6)	0.61 (0.32–1.15)
Not meeting guidelines (< 7 or > 9 h of sleep) (Ref)	288 (54.0)	189 (52.4)	68 (63.0)	1	31 (48.4)	1
Shiftwork experience						
1–5 years (Ref)	188 (35.3)	137 (38.0)	31 (28.7)	1	20 (31.3)	1
6–15 years	227 (42.6)	151 (41.8)	41 (38.0)	0.86 (0.50–1.45	35 (54.7)	1.33 (0.64–2.75)
16–30 years	88 (16.5)	51 (14.1)	29 (26.9)	0.38 (0.18–0.80) *	8 (12.5)	0.47 (0.15–1.50)
30 + years	30 (5.6)	22 (6.1)	7 (6.5)	0.69 (0.21–2.23)	1 (1.6)	0.26 (0.02–2.88)

RRR: Relative Risk Ratio; *p < 0.05

## Discussion

This study explored how sleep, diet, and physical activity (as the three pillars of health) are prioritised by Australian adults across two cohorts and the association with health behaviour correlates. Overall, diet was prioritised first by a majority of the Australian adult sample, whereas sleep was prioritised first by a majority of the shiftworker-only sample.

For Australian adults, prioritising diet was associated with increased fruit consumption (greater likelihood of reporting ≥ 2 serves per day, meeting the Australian recommendations) and decreased fast food consumption (greater likelihood of reporting less than < 2 serves of fast food per week), compared to prioritising sleep. This finding suggests a link between behaviour prioritisation and actual behaviour for diet [63], which supports the notion that, for dietary behaviour, motivation (i.e. prioritising diet) can influence behaviour (i.e. decreased fast food consumption) [64]. Conversely, these findings may support the notion that, actual behaviour influenced participant prioritisation, with those that considered themselves as having a good diet choosing diet as their prioritised health behaviour when prompted. This follows a positive feedback loop as explained by the COM-B model of behaviour, with engaging in a behaviour increasing motivation to engage in the behaviour [47]. It may also be that changes were made to diet previously to improve diet quality and while diet is no longer prioritised, the healthy behaviours have remained. Although data were not collected on the underlying motivation for the prioritisation in this study, this should be a focus of future research. Additionally, some participants chose not to answer this question, and this may be because they did not prioritise any of the health behaviours. Future research is therefore warranted into how important each behaviour is, in addition to their ranking. While work demands and work-life conflict is often reported as influencing health behaviours [40], both employed and unemployed Australians reported the same pattern of behaviour prioritisation, with diet prioritised most frequently in both groups. It may be that while both groups prioritise diet, the reasons for prioritising diet, or the barriers to achieving a good diet, are different between employed and unemployed Australians [40]. This is an important focus of future research, perhaps with qualitative data that can explore the experiences of employed and unemployed Australians with health behaviour change.

The motivation-actual behaviour relationship was not found for physical activity in the sample of Australian adults, with no association between prioritising physical activity and meeting the weekly activity recommendation of ≥ 150 min/week. The finding that prioritising physical activity does not relate to actual behaviour is in contrast to the diet findings and suggests that motivation does not always influence behaviour. This may reflect different motivations for prioritising different health behaviours, in addition to opportunities and capabilities to enact change to different health behaviours, as discussed by the COM-B model For example, one study introduced a 3-month pilot intervention targeting all aspects of the COM-B model through promoting diet and physical activity in nurses, providing opportunities to measure physical activity (pedometers and a smartphone app), and using goal setting to promote capability. From this study it was reported that diet was easier to change than to increase physical activity [65]. If physical activity is perceived as harder to change, this may explain why it was the least prioritised health behaviour in the Australian adult cohort, consistent with population data showing that a majority of adults are not meeting the physical activity guidelines in Australia [1]. The physical activity threshold that was considered in this study was meeting the minimum guidelines for MPVA as this is associated with health benefits compared to not meeting this guideline [1]. As physical activity was examined using a dichotomous variable, it is unclear how different levels of physical activity may have influenced the results (e.g., individuals reporting some activity but not meeting the recommended amount, or individuals reporting more activity than the recommended amount). However, it may be that certain populations with greater physical activity, for example athletes, are more likely to prioritise their physical activity over diet and sleep. This also follows the COM-B model, such that those that engage in a behaviour at a high level are more motivated to engage in that behaviour again in the future. Understanding behaviour prioritisation in specific population groups is an important focus of future research, particularly to ensure that the other health behaviours are not neglected.

A majority of the sample of Australian adults were not meeting the Australian sleep recommendations (7–9 h per night) [51], including those individuals that prioritised sleep. This provides more evidence to suggest that prioritisation may not lead to actual behaviour for all health behaviours. While some individual sleep behaviours were analysed in the current paper, external factors can influence sleep duration including family, friends, work schedules, and outside demands [66–68]. It may be that Australian adults perceive low level of control over their sleep habits due to external factors and consequently do not prioritise sleep despite obtaining inadequate sleep. As with physical activity, this could reflect how perceived capability influences behaviour, with sleep potentially perceived as a challenging behaviour to improve. Future research could investigate the potential external barriers influencing sleep, for example, by using the Sleep Locus of Control Scale [69]. Additionally, given the relationship between sleep and diet [41, 42, 70, 71], improving sleep duration could be promoted as part of a strategy to improve diet given this is the health behaviour that is currently prioritised.

In the sample of shiftwork-only Australians, sleep was the most prioritised health behaviour, and this was seen in each of the shiftworking industries sampled. This may be because it is well known that shiftwork over a prolonged period has a pronounced, negative impact on sleep [72], with shiftworkers reporting inadequate sleep as a major problem across multiple studies [26, 73]. Prioritising this behaviour first may reflect an understanding of this problem in this cohort of shiftworkers. Further, in previously published findings from the current dataset of shiftwork-only Australian adults, 53.9% of the sample reported having heard of sleep hygiene, indicating some understanding of sleep hygiene [26] compared to a sample of shiftworking paramedics who reported little to no understanding of sleep hygiene [74]. However, despite understanding sleep hygiene principles, the majority of the sample did not meet the recommended guidelines for adequate sleep duration. This discrepancy in prioritisation and actual behaviour is consistent with the findings for sleep in the Australian adult sample, and is perhaps a reflection of the barriers to attaining adequate sleep that shiftworkers contend with due to work schedules (e.g. long hours, inconsistent wake and bedtimes, minimal breaks, and changing rosters [72]).

In the shiftwork-only sample, shiftwork experience was associated with prioritising sleep first. Those that prioritised sleep first were more likely to have 16–30 years of shiftwork experience compared to those who prioritised diet first. Participants with 16–30 years of shiftwork experience are likely to have been exposed to chronic sleep loss due to working shiftwork schedules over a prolonged period [75, 76], and this may be why they are more likely to prioritise sleep. Together, this suggests that motivation may not be the barrier to sleep health in shiftworkers, as a majority of the sample prioritised sleep despite obtaining an inadequate amount of sleep. Intervention efforts should consider improving the opportunity and capabilities that shiftworkers have to obtain adequate sleep, rather than motivation, particularly the external influences on sleep in this population. New guidelines for healthy sleep practices for shiftworkers have been published, with guidelines for behaviours such as napping, bedtime routines, and sleep environments [77]. Interventions utilising these guidelines should be a focus of future research. While studies have investigated sleep-related interventions in shiftworkers, the intervention type is heterogenous across the literature with mixed effectiveness for improving fatigue [78]. More studies are needed to develop a comprehensive sleep intervention that incorporates the capabilities, opportunities, and motivations of shiftworkers. One way to do this is to individualise sleep interventions for shiftworkers to take into account these specific capabilities, opportunities, and motivations. A recent study has demonstrated effectiveness of an individualised sleep and shiftwork education program in nurses on anxiety and insomnia scores [79] Further, increased focus on diet and physical activity as health behaviours that shiftworkers may have more control over may be beneficial.

The two cohorts included in this study prioritised health behaviours differently. The cohorts were different as the sample of Australian adults included some shiftworkers as well as other work types and people who weren’t employed. In addition to the make-up of the cohorts, the timing of the surveys was different. The National Social Survey 2017 survey was conducted prior to the COVID-19 pandemic, whereas the shiftwork study was conducted in 2021, during the pandemic. A recent review found that during the COVID-19 pandemic, the prevalence of sleep problems was high, with approximately 40% of the population impacted [78]. This is likely due to later bedtimes and waketimes or a reduction in night-time sleep [80]. Diet and physical activity were also impacted during COVID-19, with reductions to leisure-time physical activity and increases in sedentary behaviour [81], and changes to habitual dietary patterns [82]. While physical activity and diet were not assessed in Study 2, it may be that the changes to sleep, diet, and physical activity during COVID-19 contributed to different behaviour prioritisation for these participants.

To the best of our knowledge, this study is the first to explore the prioritisation of the three pillars of health in Australian adults and in shiftworkers. Therefore, findings from this study contribute to the literature on health behaviour change for populations at-risk for chronic diseases and inform the development of policies to promote behaviour change. However, there are several limitations of the current study to consider when interpreting the results. First, both studies included in this manuscript were cross-sectional and self-report and thus subject to self-report bias and inability to determine causal relationships. The limitations of self-report data are important to consider, for example self-report dietary measures are prone to the under-reporting of energy intake, and this varies based on body mass index and can be largely influenced by social desirability [83]. Second, given the cross-sectional nature of both studies, behaviour change over time was not assessed. Sleep, diet and physical activity behaviours are likely to change daily [49], as well as across the lifespan [13].There were key differences in the methodology of each study that limit direct comparison between the two studies. These differences include no questions on diet and physical activity in the shiftwork study, which means that whether these behaviours relate to prioritising diet or physical activity in this sample is unknown. Given the increasing understanding of the importance of diet [27, 84] and physical activity [85, 86] in shiftworkers, these behaviours are important to measure in future research. Further, the National Social Survey 2017 had questions on employment status and industry, but not whether participants were shiftworkers or not. Therefore, it is likely that the sample of Australian adults included participants working shiftwork and other work patterns, but we are unable to distinguish them specifically. The shiftworking population was majority female participants, which does not reflect Australian data that suggests there are more male shiftworkers than females. This likely reflects the high proportion of the sample working in the healthcare and social assistance industry, which are majority female. The results from this project may therefore be more generalisable to shiftworking females than males, and this is a consideration for recruitment for future research.

To build on the current findings there are multiple suggestions for future research, such as assessing longitudinal changes in health behaviours and the prioritisation of the three pillars of health. Further, future research should utilise qualitative methods to understand barriers, feasibility, and acceptability of behaviour change related to the pillars of health. Additionally, qualitative research could explore other factors, both internal and external, that influence health behaviours in both shiftworkers and non-shiftworkers. These factors will be important to consider in any behavioural intervention. What health behaviours people feel satisfied would also help to provide context on the underlying motivation for ranking those behaviours. Research on health behaviours should also continue to use samples of shiftworkers and non-shiftworkers, given shiftworkers are an at-risk population for chronic disease and experience unique organisational challenges that lead to circadian disruption.

This study explored how sleep, diet, and physical activity are prioritised by Australian adults across two cohorts and how the ranking was associated with health behaviour correlates. While prioritising diet was associated with healthier diet behaviour in Australian adults, overall, across both cohorts, behaviour prioritisation did not relate to actual behaviour. These findings suggest that behaviour prioritisation is only part of the story of behaviour change and future research should focus on comprehensive approaches to behaviour change promotion that target the barriers to specific behaviours.

## Electronic supplementary material

Below is the link to the electronic supplementary material.

Supplementary Material 1

## Data Availability

The datasets used and/or analysed during the current study are available from the corresponding author on reasonable request.
